# Prognostic Implication of Small Dense LDL-Cholesterol Levels following Acute Coronary Syndrome

**DOI:** 10.3390/medicina59010158

**Published:** 2023-01-12

**Authors:** Teruhiko Imamura, Masakazu Hori, Nikhil Narang, Hiroshi Ueno, Koichiro Kinugawa

**Affiliations:** 1 The Second Department of Internal Medicine, University of Toyama, Toyama 930-0194, Japan; 2 Advocate Christ Medical Center, Oak Lawn, IL 60453, USA

**Keywords:** dyslipidemia, coronary artery disease, cardiovascular disease

## Abstract

*Background and Objectives*: Small dense LDL cholesterol is a strong risk factor for atherosclerosis. However, few studies have investigated the impacts of this specific lipid profile on the incident risk of adverse cardiovascular events in patients with acute coronary syndrome. *Materials and Methods*: Patients with acute coronary syndrome, who underwent revascularization, were included and followed for 2 years. The levels of small dense LDL cholesterol were measured at index discharge (day 0) in the setting of newly administered therapies for secondary prevention, including aspirin and statins, during the index hospitalization. The prognostic impact of small dense LDL-cholesterol levels on the risk of a primary composite endpoint, including cardiac death, non-fatal myocardial infarction, unstable angina pectoris, stroke, and heart failure, was investigated. *Results*: In total, 46 patients (median 75 (59, 83) years old, 63% men) were included. Median small dense LDL cholesterol was 19.4 (13.5, 23.8) mg/dL at index discharge. All patients initiated statin treatment before the index discharge, with a median LDL-cholesterol level of 77 (64, 109) mg/dL. Small dense LDL-cholesterol level was independently associated with an incremental risk for the primary endpoint (*p* < 0.05 by adjusting for several potential risk factors, including LDL cholesterol) with a cutoff of 32.6 mg/dL. *Conclusions*: Small dense LDL-cholesterol level was a significant risk factor for cardiovascular events following presentations of acute coronary syndrome.

## 1. Background

Small dense low-density lipoprotein (LDL) cholesterol is incorporated into clinical risk-assessment tools in primary/secondary prevention measures for ischemic heart disease [1,2]. Small dense LDL cholesterol has a large gravity but remains a small particle among LDL-cholesterol subtypes, including large, medium, and small dense LDL cholesterol [3]. The formation of small dense LDL cholesterol is accelerated, particularly among those with insulin resistance and hypertriglyceridemia [4]. Small dense LDL cholesterol is an independent risk factor for cardiovascular conditions, irrespective of baseline LDL cholesterol levels [5]. Small dense LDL cholesterol is also strongly associated with accelerated atherosclerosis, due to less affinity for LDL receptors, more increased endothelial cell adhesion, and greater vulnerability to oxidization, as compared to common LDL cholesterol [6].

The data on incident risk of small dense LDL cholesterol has primarily been reported among cohorts of patients with stable coronary artery disease [5,7,8,9,10,11,12], whereas the prognostic impact of small dense LDL-cholesterol levels among those with acute coronary syndrome remains uncertain [13,14]. More data on strategies to enhance secondary prevention of cardiovascular events among those with acute coronary syndrome are needed to prevent downstream events and further improve clinical outcomes following acute coronary syndrome.

In this study, we aimed to investigate the impact of small dense LDL cholesterol, which was measured at index discharge following the revascularization for acute coronary syndrome with the initiation of lipid-lowering medications, on the incident risk of major cardiovascular events (MACEs).

## 2. Methods

### 2.1. Patient Selection

Patients, who were hospitalized at our institute with a diagnosis of acute coronary syndrome between June 2016 and May 2021 were screened for study inclusion. The presence of acute coronary syndrome was determined by symptom assessment, echocardiography, electrocardiogram, and laboratory data in the emergency room. The diagnosis of acute coronary syndrome was confirmed by the existence of culprit lesion in the coronary angiography. When patients’ attending cardiologist was a data curator (M.H.), patients were considered to be included in this study and their small dense LDL-cholesterol levels were measured at the index discharge. All patients were followed for 2 years or until l5 October 2022. Written informed consent was obtained from all participants before the listing. The institutional review board approved the study protocol.

### 2.2. Study Design

The time of index discharge was defined as day 0. The impact of small dense LDL-cholesterol levels at discharge on the risk of meeting the primary endpoint was investigated.

### 2.3. Coronary Revascularization

Coronary angiography was performed via radial approach, in principle, by board-certified cardiologists using a standard procedure. The therapeutic strategy regarding target vessels and possibility of surgical intervention was discussed by the multidisciplinary heart team, and appropriate percutaneous coronary intervention or coronary artery bypass grafting was performed based on those specific discussions.

### 2.4. Biomarker Measurement

Lipid parameters, including small dense LDL cholesterol, were assayed using standard laboratory procedures. All serum samples were obtained on admission before heparin infusion and at the index discharge without heparinization and frozen at −80 degrees immediately. The level of small dense LDL cholesterol was measured at the external laboratory institute (BML, Inc., Tokyo, Japan).

### 2.5. Other Clinical Data Obtained

Demographics, baseline comorbidities, echocardiography, and other standard laboratory data points were obtained on admission. Procedure-related data were retrieved. Standard laboratory and medication data at index discharge were obtained.

### 2.6. Follow-Up Data

The primary endpoint was defined as the occurrence of MACEs consisting of cardiac death, non-fatal myocardial infarction, unstable angina pectoris, heart failure, and stroke, during the observational period from time of index discharge (day 0).

### 2.7. Statistical Analysis

Continuous data were displayed as median and interquartile range, irrespective of their distribution. Categorical data were displayed as numbers and percentages. Correlation between small dense LDL cholesterol and triglyceride to high-density lipoprotein cholesterol ratio was assessed by Pearson’s correlation coefficient.

The prognostic impact of small dense LDL-cholesterol level at the index discharge upon the primary endpoint was investigated by Cox proportional hazard ratio regression analyses, which was adjusted for several potential risk factors, including age, sex, max creatinine kinase, plasma B-type natriuretic peptide, and LDL cholesterol. A cutoff of small dense LDL cholesterol to predict the primary endpoint was calculated using receiver operating characteristic analysis. The cumulative incidence of the primary endpoint was stratified by the cutoff of small dense LDL cholesterol and compared between the two groups using log-rank test. Event rates of the primary endpoints during the observational period were compared between the two groups using negative binomial regression analysis.

A *p* < 0.05 was assumed statistically significant. Statistical analyses were conducted using SPSS Statistics 22 (SPSS Inc., Armonk, IL, USA).

## 3. Results

### 3.1. Baseline Characteristics on Admission

In total, 46 patients were included (Table 1). Median age was 75 (59, 83) years old and 63% of the sample was men. Plasma B-type natriuretic peptide level on admission was 108 (41, 561) pg/mL.

All patients were diagnosed with ACS. Most of them (61%) were acute myocardial infarction (Table 2). Predominant target vessel (50%) was left anterior descending artery. Most of the patients (91%) received percutaneous coronary intervention and others (9%) received coronary artery bypass grafting. Maximum creatinine kinase was 2151 (533, 7091) U/L during the index hospitalization. All interventions to treat ACS were performed during the acute phase prior to the index discharge.

### 3.2. Clinical Data at the Index Discharge

**Figure 1:**
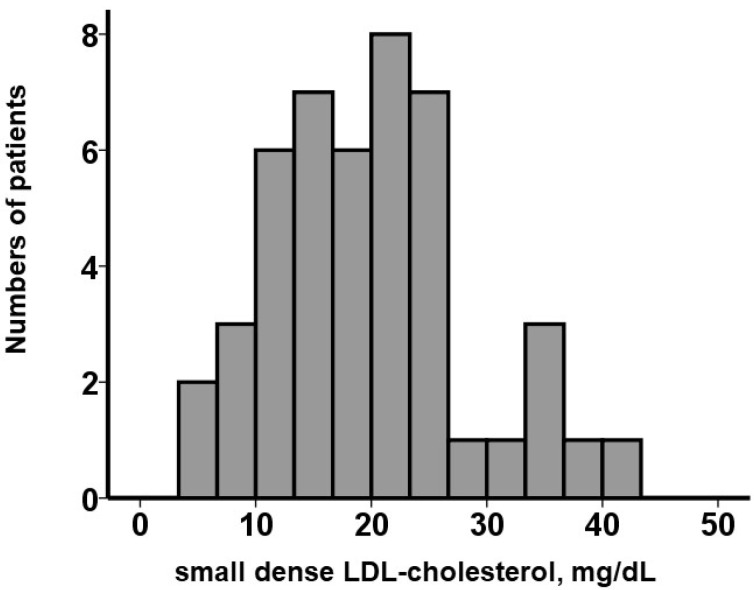
Distribution of small dense LDL cholesterol at index discharge (*N* = 46). LDL, low-density lipoprotein.

Small dense LDL-cholesterol levels were measured in all patients at index discharge, as detailed in the Methods section. Small dense LDL cholesterol was distributed widely, with a median value of 19.4 (13.5, 23.8) mg/dL (Figure 1).

Other laboratory and medication data are displayed in Table 3. LDL-cholesterol level was 77 (64, 109) mg/dL and triglyceride level was 109 (74, 140) mg/dL. Triglyceride to high-density lipoprotein cholesterol ratio and small dense LDL cholesterol had a mild correlation (r = 0.300, *p* = 0.043). All patients received strong statins (atorvastatin, losbastatin, or pitavastatin) and 10 patients (22%) received ezetimibe. Only one patient received fenofibrate.

### 3.3. Prognostic Impact of Small Dense LDL-Cholesterol Levels at the Index Discharge

Following index discharge, patients were followed for 681 (495, 730) days. During the observational period, the primary endpoint occurred in seven patients consisting of three episodes of worsening angina that required coronary interventions, two episodes of acute coronary syndrome, and three episodes of worsening heart failure. Of note, seven out of nine events occurred during the 1-year follow-up.

Small dense LDL cholesterol was a risk factor for future MACE following adjustment for several potential risk factors (*p* < 0.05, Table 4). Of note, its significance remained following the adjustment for LDL cholesterol (*p* = 0.021). The prognostic impact of small dense LDL cholesterol remained significant among those with percutaneous coronary intervention alone (*N* = 42) (hazard ratio 1.17, 95% confidence interval 1.06–1.29, *p* = 0.002).

**Figure 2:**
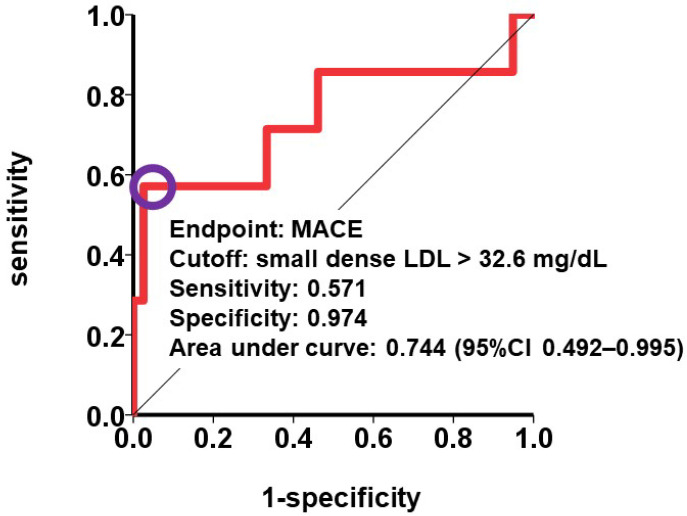
A cutoff of small dense LDL cholesterol to predict incidence of MACE. LDL, low-density lipoprotein; MACE, major adverse cardiac event.

**Figure 3:**
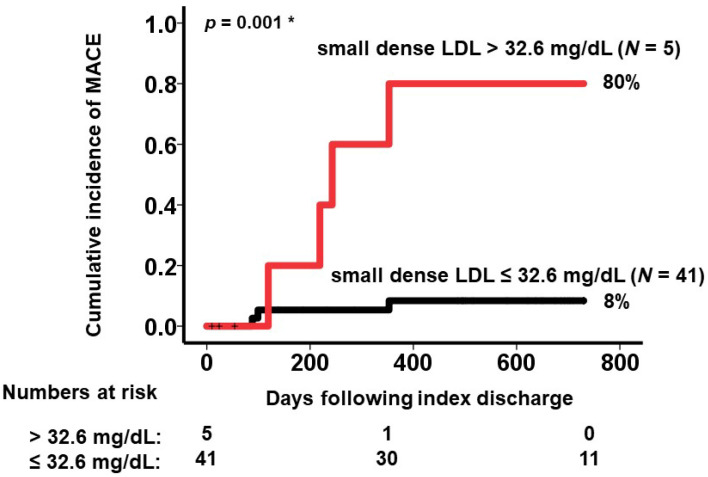
Cumulative incidence of MACE stratified by the level of small dense LDL cholesterol at index discharge. LDL, low-density lipoprotein; MACE, major adverse cardiac event. * *p* < 0.05 by log-rank test.

**Figure 4:**
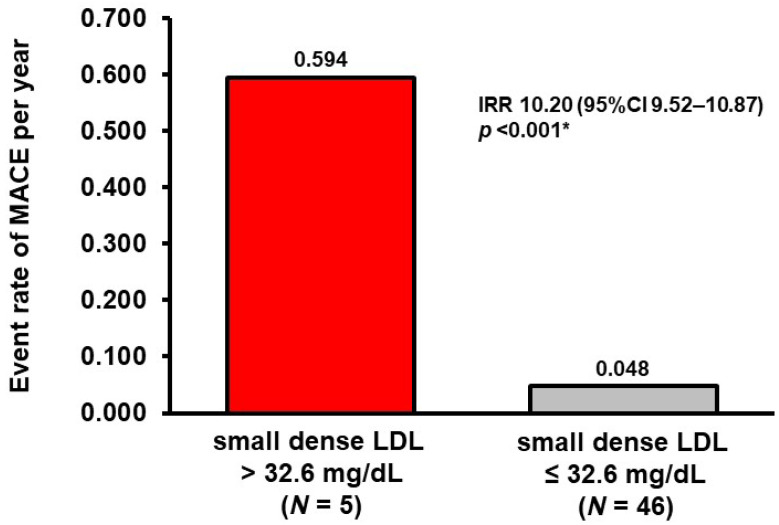
The rate of MACE during the observational period stratified by the level of small dense LDL cholesterol. * *p* < 0.05 by negative binomial regression analysis. MACE, major adverse cardiac event; LDL, low-density lipoprotein; IRR, incidence rate ratio.

A cutoff to predict MACE was calculated as 32.6 mg/dL with a sensitivity of 0.571 and a specificity of 0.974 (Figure 2). Five patients had small dense LDL cholesterol >32.6 mg/dL. They had a significantly higher cumulative incidence of the primary endpoint compared with those with small dense LDL cholesterol ≤32.6 mg/dL (80% versus 8%, *p* = 0.001; Figure 3). The event rate of MACE per year was significantly higher in the patients with small dense LDL cholesterol >32.6 mg/dL (incidence rate ratio 10.20, *p* < 0.001: Figure 4).

### 3.4. Clinical Data Follow-Up

At index discharge, laboratory data were not significantly differentiated by small dense LDL-cholesterol level, except for high-density lipoprotein cholesterol levels (Table 5). Of note, triglyceride to high-density lipoprotein cholesterol ratio tended to be higher in patients with higher small dense LDL cholesterol (*p* = 0.16). Such trends remained at the three-month follow-up (*p* = 0.084). Remnant cholesterol was not correlated with small dense LDL cholesterol (r = 0.27, *p* = 0.27) but had a significant correlation with triglyceride to high-density lipoprotein cholesterol ratio (r = 0.71, *p* < 0.001).

## 4. Discussion

In this study, we investigated the impact of small dense LDL-cholesterol level, which was measured at index discharge following a diagnosis of acute coronary syndrome and the initiation of lipid-lowering medications, upon post-discharge occurrence of MACE. The higher small dense LDL-cholesterol level with a cutoff of 32.6 mg/dL was associated with the incremental incidence of MACE.

### 4.1. Impact of Small Dense LDL Cholesterol on the Occurrence of MACE

The prognostic impact of small dense LDL-cholesterol level upon the sub-clinical progression of coronary artery atherosclerosis [9,10], the occurrence of stable coronary artery disease [5,7,8], and the occurrence of cardiovascular disease [15] has been observed in healthy cohorts in multiple recent studies. Of note, the prognostic impact of small dense LDL cholesterol on the incident risk of coronary heart disease remained significant, even among those with well-controlled LDL cholesterol <100 mg/dL in patients studied in the atherosclerosis risk in communities (ARIC) study [5]. Other studies have investigated the prognostic impact of small dense LDL cholesterol among those with a history of coronary artery disease [11,12] and observed an increased risk of adverse cardiovascular events with an incremental rise in small dense LDL cholesterol among those with stable coronary disease.

Few studies investigated the prognostic impact of small dense LDL cholesterol among those with acute coronary syndrome, as examined in this study. In a study conducted by Zhang and colleagues, patients with elevated small dense LDL-cholesterol levels on admission had a higher risk of cardiovascular events in those with acute coronary syndrome receiving percutaneous coronary intervention [14]. We did not assess small dense LDL-cholesterol levels on admission as the trend in their levels should be determined after initiation of lipid-lowering agents that can also affect small dense LDL-cholesterol levels [16], most of which are initiated during index hospitalization. Thus, we measured small dense LDL-cholesterol levels at index discharge. In another study, including those with acute coronary syndrome, small dense LDL-cholesterol levels that were measured downstream at 10 months following initial presentation were associated with disease progression in non-culprit vessels from initial coronary angiogram [13].

Our study offers new insight into understanding this marker as a predictor of incident risk with measurement at the time of index discharge in the background of lipid-lowering therapy. Of note, the prognostic impact of small dense LDL-cholesterol level was independent of LDL-cholesterol levels also in this cohort.

### 4.2. Future Directions

Despite the available data, the measurement of small dense LDL cholesterol has not been conducted in Japan. According to previous studies, triglyceride to high-density lipoprotein–cholesterol ratio might be a good surrogate of small dense LDL cholesterol [17].

Given the small sample size, we could not derive robust findings about the clinical characteristics and trends in clinical parameters of those with high small dense LDL cholesterol, which should further clarify the underlying mechanism and explanation of why high small dense LDL cholesterol had a negative prognostic impact. Association between small dense LDL cholesterol and comorbidities, such as metabolic syndrome and insulin resistance, remains a future concern [4]. The impact of persistently elevated small dense LDL-cholesterol levels against optimal revascularization and medications, including anti-platelets and lipid-lowering therapies, on various cardiovascular parameters also remains a concern [18,19].

Another area needing further study is what medical interventions in the setting of elevated small dense LDL cholesterol may have an impact on reducing the risk of future MACE. Pemafibrate is a selective peroxisome proliferator-activated receptor-α modulator, which has been shown to reduce serum triglyceride levels with few drug-related adverse events [20]. The impact of pemafibrate to modulate small dense LDL-cholesterol levels and reduce the risk of MACE is an area needing further investigation [21]. Interventions for metabolic syndrome, diabetic mellitus, and obesity might be another promising strategy to lower small dense LDL-cholesterol levels and reduce the risk of MACE [22].

### 4.3. Limitations

This study includes a small sample cohort. Given the small event numbers, we could not include many potential confounders in the multivariable analyses. This is a proof-of-concept study. Further studies consisting of multi-institution participation are warranted to validate our findings. Our cohort was heterogeneous, including a variety of severity and procedures (percutaneous coronary intervention and coronary artery bypass grafting), which might also have affected the clinical outcomes. We measured small dense LDL-cholesterol levels only one time at index discharge. Instead, we followed other lipid parameters, including triglyceride to high-density lipoprotein cholesterol ratio, as an alternative marker of small dense LDL cholesterol. The clinical implication of its trend needs further assessment.

## 5. Conclusions

Small dense LDL-cholesterol level was a significant risk factor for cardiovascular events following acute coronary syndrome, even with statin therapy and relatively well-controlled LDL-cholesterol and triglyceride levels.

## Figures and Tables

**Table 1:** Baseline characteristics on admission.

	*N* = 46
*Demographics*	
Age, years	75 (59, 83)
Men	29 (63%)
Body mass index	23.0 (21.1, 25.7)
*Comorbidity*	
Diabetes mellitus	19 (41%)
Atrial fibrillation	5 (11%)
History of stroke	7 (15%)
History of heart failure	4 (9%)
*Echocardiography*	
Left ventricular end-diastolic diameter, mm	45 (40, 49)
Left ventricular ejection fraction, %	53 (33, 60)
Moderate or greater mitral regurgitation	1 (2%)
Moderate or greater tricuspid regurgitation	4 (9%)
*Laboratory data*	
Estimated glomerular filtration ratio, mL/min/1.73m^2^	60.9 (34.5, 76.0)
Plasma B-type natriuretic peptide, pg/mL	108 (41, 561)
HDL-cholesterol, mg/dL	47 (41, 59)
LDL-cholesterol, mg/dL	93 (76, 124)
Triglyceride, mg/dL	82 (56, 133)
*Medication*	
Loop diuretics	4 (9%)
Beta-blockers	9 (20%)
Renin-angiotensin system inhibitors	19 (41%)
Mineralocorticoid receptor antagonists	3 (7%)
SGLT2 inhibitors	4 (9%)
Statin	14 (30%)
Fibrate	1 (2%)
Ezetimibe	0 (0%)

Continuous data are stated as median and interquartile. Categorical data are stated as numbers and percentage.

**Table 2:** Procedure characteristics.

	*N* = 46
*Type of disease*	
Acute myocardial infarction	28 (61%)
Recent myocardial infarction	4 (9%)
Unstable angina pectoris	14 (30%)
*Target vessel*	
Left main trunk	6 (13%)
Left anterior descending artery	23 (50%)
Right coronary artery	11 (24%)
Left circumflex artery	6 (13%)
*Procedure*	
Percutaneous coronary intervention	42 (91%)
Coronary artery bypass grafting	4 (9%)
*Maximum creatinine kinase, U/L*	2151 (533, 7091)
*Days of hospitalization, days*	16 (10, 32)

Continuous data are stated as median and interquartile. Categorical data are stated as numbers and percentage.

**Table 3:** Clinical characteristics at the index discharge.

	*N* = 46
*Laboratory data*	
Estimated glomerular filtration ratio, mL/min/1.73m^2^	61.3 (52.4, 74.0)
Plasma B-type natriuretic peptide, pg/mL	140 (73, 290)
HDL-cholesterol, mg/dL	42 (35, 48)
LDL-cholesterol, mg/dL	77 (64, 109)
Triglyceride, mg/dL	109 (74, 140)
Triglyceride to HDL-cholesterol ratio	2.41 (1.32, 3.48)
*Medication*	
Loop diuretics	11 (24%)
Beta-blockers	36 (78%)
Renin-angiotensin system inhibitors	37 (80%)
Mineralocorticoid receptor antagonists	12 (26%)
SGLT2 inhibitors	12 (26%)
Statin	46 (100%)
Fibrate	1 (2%)
Ezetimibe	10 (22%)

Continuous data are stated as median and interquartile. Categorical data are stated as numbers and percentage. HDL, high-density lipoprotein; LDL, low-density lipoprotein; SGLT2, sodium-glucose cotransporter.

**Table 4:** Impact of small dense LDL cholesterol at the index discharge upon the primary endpoint.

	Hazard Ratio	95% Confidence Interval	*p* Value
*Univariable*			
Small dense LDL cholesterol	1.114	1.026–1.209	0.010 *
*Multivariable*			
Small dense LDL cholesterol + age	1.125	1.029–1.231	0.010 *
Small dense LDL cholesterol + men	1.112	1.024–1.208	0.012 *
Small dense LDL cholesterol + max creatinine kinase	1.114	1.026–1.209	0.010 *
Small dense LDL cholesterol + BNP	1.174	1.054–1.308	0004 *
Small dense LDL cholesterol + LDL cholesterol	1.114	1.016–1.222	0.021 *

* *p* < 0.05 by Cox proportional hazard ratio regression analyses. LDL, low-density lipoprotein; BNP, B-type natriuretic peptide.

**Table 5:** Laboratory data follow-up.

	Small Dense LDL >32.6 (*N* = 5)	Small Dense LDL ≤32.6 (*N* = 41)	*p* Value
*At index discharge*			
Estimated glomerular filtration ratio, mL/min/1.73 m^2^	58.9 (41.8, 65.1)	61.7 (52.4, 76.5)	0.49
Plasma B-type natriuretic peptide, pg/mL	101 (77, 154)	160 (60, 327)	0.66
HDL-cholesterol, mg/dL	35 (30, 40)	43 (36, 50)	0.042 *
LDL-cholesterol, mg/dL	79 (61, 106)	75 (61, 96)	0.15
Remnant cholesterol, mg/dL	35 (9, 40)	21 (15, 27)	0.43
Triglyceride, mg/dL	185 (126, 230)	107 (69, 140)	0.21
Triglyceride to HDL-cholesterol ratio	4.52 (2.28, 5.00)	2.40 (1.32, 3.20)	0.16
*Three months follow-up*			
Estimated glomerular filtration ratio, mL/min/1.73 m^2^	56.7 (40.1, 63.4)	61.2 (52.1, 75.6)	0.41
Plasma B-type natriuretic peptide, pg/mL	142 (98, 243)	121 (43, 223)	0.45
HDL-cholesterol, mg/dL	33 (28, 38)	45 (37, 52)	0.037 *
LDL-cholesterol, mg/dL	77 (60, 102)	76 (60, 97)	0.43
Remnant cholesterol, mg/dL	37 (11, 42)	20 (14, 26)	0.18
Triglyceride, mg/dL	192 (131, 245)	103 (66, 137)	0.13
Triglyceride to HDL-cholesterol ratio	4.77 (2.34, 5.14)	2.34 (1.21, 3.14)	0.084

Continuous data are stated as median and interquartile. HDL, high-density lipoprotein; LDL, low-density lipoprotein. * *p* < 0.05 by Mann–Whitney U test.

## Data Availability

Data set is available upon reasonable request.

## References

[1] Ivanova E.A., Myasoedova V.A., Melnichenko A.A., Grechko A.V., Orekhov A.N. (2017). Small Dense Low-Density Lipoprotein as Biomarker for Atherosclerotic Diseases. Oxid. Med. Cell. Longev..

[2] Vekic J., Zeljkovic A., Cicero A.F.G., Janez A., Stoian A.P., Sonmez A., Rizzo M. (2022). Atherosclerosis Development and Progression: The Role of Atherogenic Small, Dense LDL. Medicina.

[3] Hayashi T., Koba S., Ito Y., Hirano T. (2017). Method for estimating high sdLDL-C by measuring triglyceride and apolipoprotein B levels. Lipids Health Dis..

[4] Hirano T. (2018). Pathophysiology of Diabetic Dyslipidemia. J. Atheroscler. Thromb..

[5] Hoogeveen R.C., Gaubatz J.W., Sun W., Dodge R.C., Crosby J.R., Jiang J., Couper D., Virani S.S., Kathiresan S., Boerwinkle E. (2014). Small dense low-density lipoprotein-cholesterol concentrations predict risk for coronary heart disease: The Atherosclerosis Risk In Communities (ARIC) study. Arterioscler. Thromb. Vasc. Biol..

[6] McPherson R. (2013). Remnant cholesterol: “Non-(HDL-C + LDL-C)” as a coronary artery disease risk factor. J. Am. Coll. Cardiol..

[7] St-Pierre A.C., Cantin B., Dagenais G.R., Mauriege P., Bernard P.M., Despres J.P., Lamarche B. (2005). Low-density lipoprotein subfractions and the long-term risk of ischemic heart disease in men: 13-year follow-up data from the Quebec Cardiovascular Study. Arterioscler. Thromb. Vasc. Biol..

[8] Higashioka M., Sakata S., Honda T., Hata J., Yoshida D., Hirakawa Y., Shibata M., Goto K., Kitazono T., Osawa H. (2020). Small Dense Low-Density Lipoprotein Cholesterol and the Risk of Coronary Heart Disease in a Japanese Community. J. Atheroscler. Thromb..

[9] Qi Y., Liu J., Wang W., Wang M., Zhao F., Sun J., Liu J., Deng Q., Zhao D. (2020). High sdLDL Cholesterol can be Used to Reclassify Individuals with Low Cardiovascular Risk for Early Intervention: Findings from the Chinese Multi-Provincial Cohort Study. J. Atheroscler. Thromb..

[10] Williams P.T., Zhao X.Q., Marcovina S.M., Otvos J.D., Brown B.G., Krauss R.M. (2014). Comparison of four methods of analysis of lipoprotein particle subfractions for their association with angiographic progression of coronary artery disease. Atherosclerosis.

[11] Jin J.L., Zhang H.W., Cao Y.X., Liu H.H., Hua Q., Li Y.F., Zhang Y., Wu N.Q., Zhu C.G., Xu R.X. (2020). Association of small dense low-density lipoprotein with cardiovascular outcome in patients with coronary artery disease and diabetes: A prospective, observational cohort study. Cardiovasc. Diabetol..

[12] Sakai K., Koba S., Nakamura Y., Yokota Y., Tsunoda F., Shoji M., Itoh Y., Hamazaki Y., Kobayashi Y. (2018). Small dense low-density lipoprotein cholesterol is a promising biomarker for secondary prevention in older men with stable coronary artery disease. Geriatr. Gerontol. Int..

[13] Sekimoto T., Koba S., Mori H., Sakai R., Arai T., Yokota Y., Sato S., Tanaka H., Masaki R., Oishi Y. (2021). Small Dense Low-Density Lipoprotein Cholesterol: A Residual Risk for Rapid Progression of Non-Culprit Coronary Lesion in Patients with Acute Coronary Syndrome. J. Atheroscler. Thromb..

[14] Zhang J., He L. (2021). Relationship between small dense low density lipoprotein and cardiovascular events in patients with acute coronary syndrome undergoing percutaneous coronary intervention. BMC Cardiovasc. Disord..

[15] Arai H., Kokubo Y., Watanabe M., Sawamura T., Ito Y., Minagawa A., Okamura T., Miyamato Y. (2013). Small dense low-density lipoproteins cholesterol can predict incident cardiovascular disease in an urban Japanese cohort: The Suita study. J. Atheroscler. Thromb..

[16] Santos H.O., Earnest C.P., Tinsley G.M., Izidoro L.F.M., Macedo R.C.O. (2020). Small dense low-density lipoprotein-cholesterol (sdLDL-C): Analysis, effects on cardiovascular endpoints and dietary strategies. Prog. Cardiovasc. Dis..

[17] Maruyama C., Imamura K., Teramoto T. (2003). Assessment of LDL particle size by triglyceride/HDL-cholesterol ratio in non-diabetic, healthy subjects without prominent hyperlipidemia. J. Atheroscler. Thromb..

[18] Ikezaki H., Lim E., Cupples L.A., Liu C.T., Asztalos B.F., Schaefer E.J. (2021). Small Dense Low-Density Lipoprotein Cholesterol Is the Most Atherogenic Lipoprotein Parameter in the Prospective Framingham Offspring Study. J. Am. Heart Assoc..

[19] Ikezaki H., Furusyo N., Yokota Y., Ai M., Asztalos B.F., Murata M., Hayashi J., Schaefer E.J. (2020). Small Dense Low-Density Lipoprotein Cholesterol and Carotid Intimal Medial Thickness Progression. J. Atheroscler. Thromb..

[20] Yamashita S., Masuda D., Matsuzawa Y. (2020). Pemafibrate, a New Selective PPARalpha Modulator: Drug Concept and Its Clinical Applications for Dyslipidemia and Metabolic Diseases. Curr. Atheroscler. Rep..

[21] Pradhan A.D., Paynter N.P., Everett B.M., Glynn R.J., Amarenco P., Elam M., Ginsberg H., Hiatt W.R., Ishibashi S., Koenig W. (2018). Rationale and design of the Pemafibrate to Reduce Cardiovascular Outcomes by Reducing Triglycerides in Patients with Diabetes (PROMINENT) study. Am. Heart J..

[22] Wu L., Parhofer K.G. (2014). Diabetic dyslipidemia. Metabolism.

